# Radiomics in the characterization of lipid-poor adrenal adenomas at unenhanced CT: time to look beyond usual density metrics

**DOI:** 10.1007/s00330-023-10090-8

**Published:** 2023-08-11

**Authors:** Giacomo Feliciani, Francesco Serra, Enrico Menghi, Fabio Ferroni, Anna Sarnelli, Carlo Feo, Maria Chiara Zatelli, Maria Rosaria Ambrosio, Melchiore Giganti, Aldo Carnevale

**Affiliations:** 1 Medical Physics Unit, IRCCS Istituto Romagnolo per lo Studio dei Tumori (IRST) “Dino Amadori”, Meldola, Italy; 2 Department of Translational Medicine - Section of Radiology, University of Ferrara, Ferrara, Italy; 3 Radiology Unit, IRCCS Istituto Romagnolo per lo Studio dei Tumori (IRST) “Dino Amadori”, Meldola, Italy; 4 Department of Medical Sciences, University of Ferrara, Ferrara, Italy; 5 Department of Medical Sciences – Section of Endocrinology and Internal Medicine, University of Ferrara, Ferrara, Italy

**Keywords:** Abdomen, Adrenocortical adenoma, Adrenal incidentaloma, X-ray computed tomography, Artificial intelligence

## Abstract

**Objectives:**

In this study, we developed a radiomic signature for the classification of benign lipid-poor adenomas, which may potentially help clinicians limit the number of unnecessary investigations in clinical practice. Indeterminate adrenal lesions of benign and malignant nature may exhibit different values of key radiomics features.

**Methods:**

Patients who had available histopathology reports and a non-contrast-enhanced CT scan were included in the study. Radiomics feature extraction was done after the adrenal lesions were contoured. The primary feature selection and prediction performance scores were calculated using the least absolute shrinkage and selection operator (LASSO). To eliminate redundancy, the best-performing features were further examined using the Pearson correlation coefficient, and new predictive models were created.

**Results:**

This investigation covered 50 lesions in 48 patients. After LASSO-based radiomics feature selection, the test dataset’s 30 iterations of logistic regression models produced an average performance of 0.72. The model with the best performance, made up of 13 radiomics features, had an AUC of 0.99 in the training phase and 1.00 in the test phase. The number of features was lowered to 5 after performing Pearson’s correlation to prevent overfitting. The final radiomic signature trained a number of machine learning classifiers, with an average AUC of 0.93.

**Conclusions:**

Including more radiomics features in the identification of adenomas may improve the accuracy of NECT and reduce the need for additional imaging procedures and clinical workup, according to this and other recent radiomics studies that have clear points of contact with current clinical practice.

**Clinical relevance statement:**

The study developed a radiomic signature using unenhanced CT scans for classifying lipid-poor adenomas, potentially reducing unnecessary investigations that scored a final accuracy of 93%.

**Key Points:**

*• Radiomics has potential for differentiating lipid-poor adenomas and avoiding unnecessary further investigations.*

*• Quadratic mean, strength, maximum 3D diameter, volume density, and area density are promising predictors for adenomas.*

*• Radiomics models reach high performance with average AUC of 0.95 in the training phase and 0.72 in the test phase.*

**Supplementary information:**

The online version contains supplementary material available at 10.1007/s00330-023-10090-8.

## Introduction

An adrenal incidentaloma is defined as an asymptomatic adrenal mass discovered on imaging that was not performed to investigate a suspected adrenal disease [1]. In most cases, adrenal incidentalomas represent benign non-functioning adenomas, but they may also correspond to different conditions requiring full clinical attention and therapeutic intervention (e.g., adrenocortical carcinoma, pheochromocytoma, hormone-producing adenoma or metastasis). As a consequence of the burgeoning use of advanced diagnostic imaging in daily medical practice, in the last decades, we have observed a constantly increasing incidence rate of incidentally discovered adrenal nodules. Indeed, adrenal incidentalomas are common, estimated to occur in approximately 3 to 7% of adults [2, 3].

Incidental adrenal masses represent diagnostic challenges for both radiologists and referring clinicians, particularly when the initial imaging features are equivocal or non-diagnostic. The main challenge is correctly identifying the infrequent unexpected malignant lesions (or hyperfunctioning adenomas), while sparing the vast majority of patients with clinically insignificant disease from unnecessary further examinations.

Diagnostic imaging is crucial in the classification of adrenal masses, since the precise etiology can be determined on both computed tomography (CT) and magnetic resonance imaging (MRI) for several entities without the need for further tests [1, 4].

In particular, CT could aid the diagnosis of adrenal adenomas in two ways, namely density measurement and contrast washout. A density lesser than 10 HU on non-enhanced CT (NECT) is almost always diagnostic of a lipid-rich adenoma, regardless of size [2]. By contrast, if there are no benign diagnostic imaging features (for instance, macroscopic fat, adrenal density <10 HU), a dedicated adrenal CT protocol including a 15-min delayed acquisition after contrast media administration is advisable, in order to assess the absolute—or relative—percentage washout. However, pheochromocytomas and adrenal metastases from hypervascular primary extra-adrenal malignancies could sometimes exhibit a washout pattern similar to that of adrenal adenomas [5–8].

Other imaging modalities may be useful to clarify the nature of the nodule, in particular MRI, in which a signal loss between in- and opposed-phase images at chemical-shift imaging is diagnostic of adenoma, or positron emission tomography (PET)-CT, in which most adenomas show FDG uptake less than 3.1 [9].

However, the need for additional tests puts patients at risk of anxiety and unnecessary harm from diagnostic procedures; additionally, the costs incurred can be significant.

Radiomics refers to a rapidly emerging discipline based on the extraction of mineable data from medical imaging. It has been used in oncology to support diagnosis, prognostication, and clinical decision-making, with the goal of delivering precision medicine [10–13].

In recent research, O’Shea et al [14] and Cao and Xu [15] demonstrated that early-stage metastases may be differentiated from lipid-poor adenomas using contrast-enhanced CT and NECT radiomics feature–based models with high performances. In other research, radiomics was used to distinguish lipid-poor adenomas from paragangliomas, phrochromocytomas, or carcinomas [16, 17].

To discriminate lipid-poor adenomas from other adrenal lesions, Zhang et al [18] recently developed three prediction models using conventional, radiomics, and combined feature nomograms. However, there was no significant difference in performance between the radiomic and traditional models.

In this study, we retrospectively assessed a dataset of adrenal masses with pathological confirmation that had been classified at NECT as indeterminate and that had not been distinguished by standard clinical demographic or radiological characteristics.

We hypothesized that indeterminate adrenal lesions of benign and malignant nature may exhibit different values of key radiomics features, and we developed a radiomic signature for the classification of benign lipid-poor adenomas, which may potentially help clinicians limit the number of unnecessary investigations in clinical practice.

## Materials and methods

This retrospective study was conducted according to the Declaration of Helsinki; local Ethics Committee approval for data collection was obtained (Ethics Committee of Area Vasta Emilia Centrale (AVEC); protocol code: 146/2022/Oss/AOUFe, approved on 17/02/2022). All investigations were performed by routine clinical practice and retrospectively retrieved.

### Population

Hospital discharge form (Scheda di Dimissione Ospedaliera – SDO) database of Sant’Anna University Hospital of Ferrara was searched to find all ICD-9-CM (International Classification of Diseases, Ninth Revision, Clinical Modification) coded interventions that included surgical resection of unilateral or both adrenal glands, between January 2003 and December 2018, independently by clinical suspicion or diagnosis.

A total of 251 patients that underwent adrenalectomy were identified.

All histopathology reports were reviewed, aiming to exclude patients with large infiltrating lesions (adrenalectomy done as “en bloc” resection with other near tissues and organs during large retroperitoneal tumor debulking) or adrenal cortical hypertrophy, thus including only focal adrenal lesions. Subjects who were missing a complete histopathologic electronic report in our Institutional Pathology Database were excluded from further evaluation. Lesions with a maximum diameter of less than 1 cm were not included in this study.

Preoperative radiologic imaging data were retrieved by querying the institutional Radiological Information Systems - Picture Archiving and Communication system (RIS-PACS; Philips VuePACS, Philips Medical Systems), and only the patients for whom a non-enhanced CT (NECT) scan was available were included.

### CT data

Each CT examination was reviewed independently by two abdominal radiologists, with 3 and 10 years of experience, respectively, who were blinded to the patients’ pathological data, in order to exclude the lesions with gross fat component or showing median attenuation less than 10 HU, by applying a single region of interest (ROI) encompassing more than 50% of the target lesion in the axial plane demonstrating the maximal lesion extent. Disagreements between the readers were resolved through consensus.

The CT studies analyzed after the application of the inclusion and exclusion criteria were acquired on 4 different multidetector scanners: Philips Brilliance 64 (Philips Medical Systems), GE Lightspeed VCT (GE Healthcare), Philips iCT 256 (Philips Medical Systems), Siemens Biograph 64 (Siemens Medical solutions).

The CT examinations were acquired using standard acquisition parameters adjusted to patients’ biometrics and accordingly to the purpose of the investigation (10–400 effective mAs, 120 kVp, 1.375–1.75 pitch, and 1.5–3 mm slice reconstruction thickness). Images were reconstructed using a standard soft tissue kernel used in clinical practice (namely for GE – standard, Philips – B. Siemens – Qr40).

### Imaging analysis and segmentation

A last-year radiology resident retrieved the CT images from the RIS-PACS database, fully anonymized and de-identified, in DICOM (Digital Imaging and Communications in Medicine) format. First-pass segmentations were manually performed by the same radiology fellow who contoured the adrenal lesions using 3DSlicer software with SlicerRT extension [19] on each axial image, finally obtaining a three-dimensional contoured volume of interest (VOI).

**Fig. 1:**
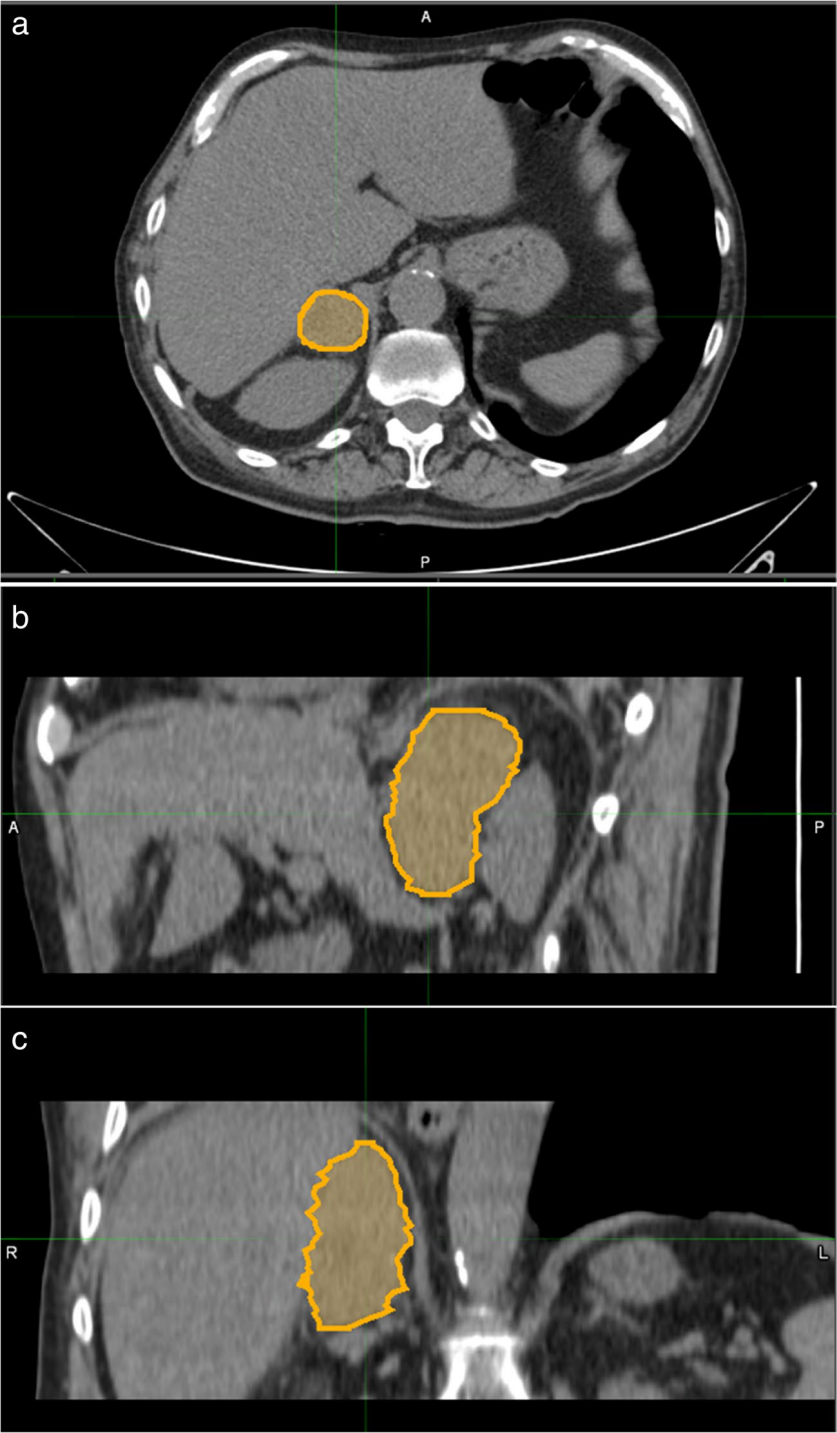
Segmentation process performed on a nodule in the right adrenal gland showed respectively in the axial (**a**), sagittal (**b**), and coronal planes (**c**)

The contoured volumes had to contain the whole adrenal mass, including the edges but avoiding the peri-adrenal soft tissues (fat, vessels, and the parenchyma of adjacent organs) (Fig. 1).

The appropriateness of the contouring process was determined by the same two experienced abdominal radiologists.

The segmented VOIs were subsequently exported as DICOM files with the RT option enabled from the SlicerRT extension.

### Image pre-processing and radiomics analysis

Following manual segmentation, images were exported to the Image Biomarker Standardization Initiative (IBSI) [16] compliant software SOPHiA DDM^TM^ Radiomics (Sophia Genetics). The patients’ CT images were resampled to a resolution of 1/1/1 mm to standardize the dataset, and grey-level quantization was performed at 32 bins prior to radiomics analysis.

Radiomics analysis software extracted 209 imaging features for each segmented volume.

Features included first-, second-, and higher-order features. The histogram of voxel intensities was employed to calculate first-order features. Intensity size-zone, co-occurrence, and run-length-based matrices were used to calculate second- and higher-order features. The IBSI Reference Handbook contains a detailed description of the 209 imaging features extracted [20].

### Statistical analysis

**Fig. 2:**
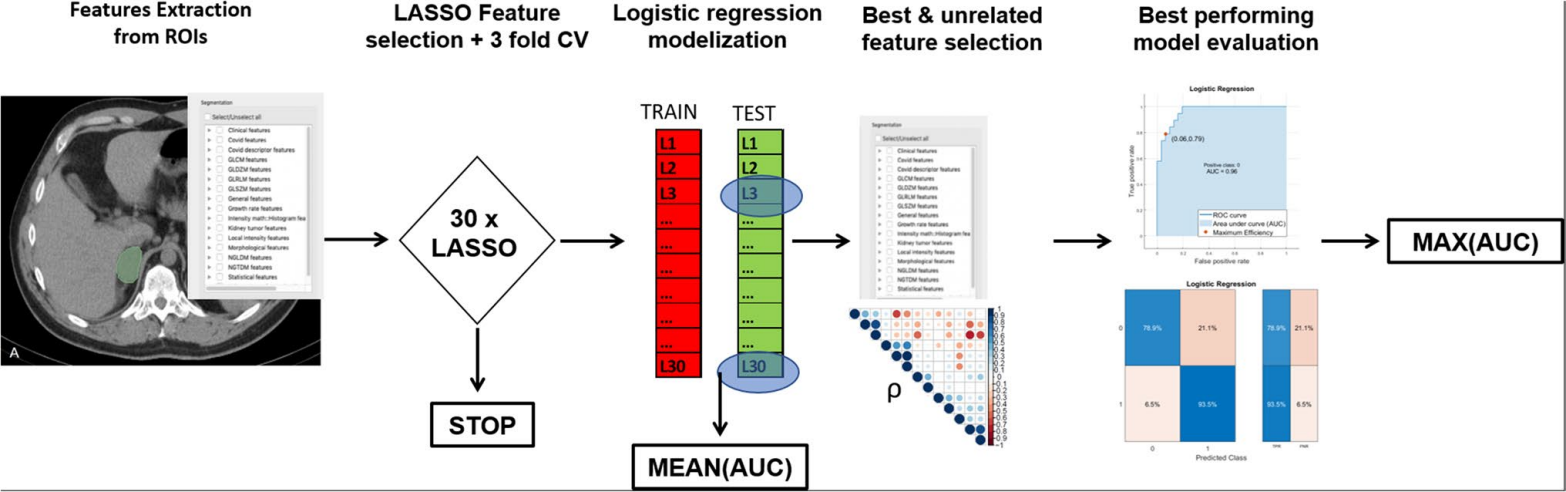
Radiomics and statistical workflow from features extraction to selection of the best-performing machine learning models

The endpoint of this study was to investigate the diagnostic performance of radiomics features extracted from patients’ CT images to differentiate between pathologically proven adenomas (labelled from now on as 0) and other adrenal histotypes (labelled from now on as 1). Clinical and demographical characteristics of the cohort were analyzed with a multivariable logistic regression for the endpoint of this study. We summarize the entire process of radiomics analysis from feature extraction to statistical model evaluations in Fig. 2.

To assess the models’ performance, we employed a stochastic cross-validation technique. The lesion feature datasets were separated into a training (2/3) and test (1/3) set during the modelling process. The training set was then used to train a logistic regression model using features picked using a least absolute shrinkage and selection operator (LASSO) technique with internal 3-fold cross-validation with the objective of maximizing the distinction between adenomas and non-adenomas.

On the test set, the predictive ability of the model was calculated. This procedure was repeated 30 times, with each iteration’s receiver operating curves (ROC) and area under the curve (AUC) being recorded for both the training and test sets. The average value of the latter ones was then used to assess the overall diagnostic performance of the model. The features that performed better in the best model of the test phase were further processed with Pearson’s *ρ* correlation coefficient to remove redundancy setting a threshold of 0.80. As the final feature selection method, we employed the selection frequency of LASSO in 30 repetitions. In the end, we built four machine learning models (logistic regression, linear discriminant, support vector machine, and decision tree) with a fixed number of lesions per feature (10 lesions per feature included in the model) to avoid overfitting the whole lesion dataset. The performance of the best model was evaluated with calibration and a decision curve to assess the consistency of the classification and its clinical usefulness.

### Data availability

Radiomics features extracted from the 50 lesions and corresponding status (adenoma/non-adenoma) that were used to develop the models are available in supplementary material 1. CT images of the patients are available upon reasonable request to the corresponding author.

## Results

### Patients’ characteristics and histopathology

**Fig. 3:**
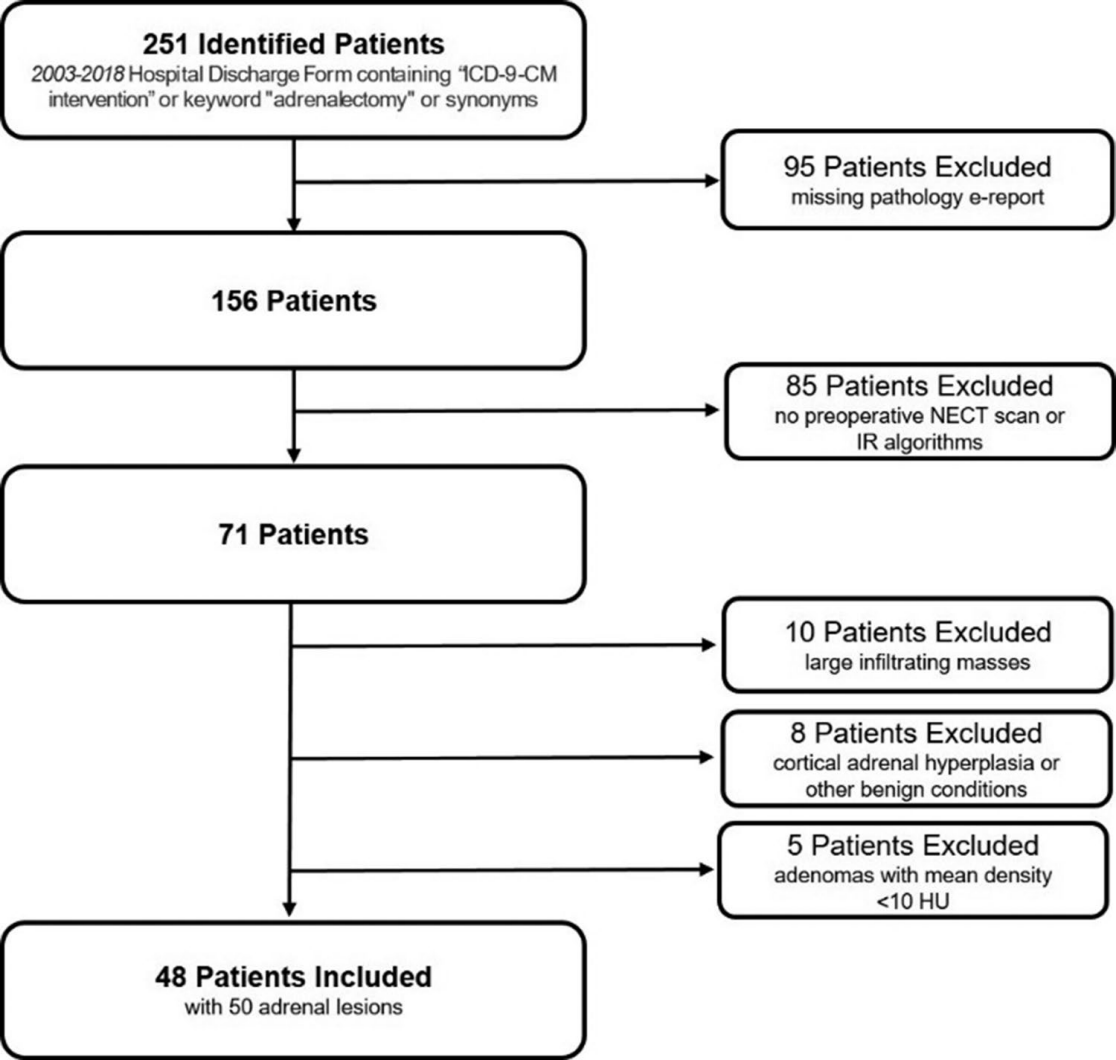
Flowchart of patients’ selection

Patients’ selection workflow is shown in Fig. 3.

The final study population consisted of 48 patients (26 males, 22 females) accounting for 50 lesions (24 in the female population, 26 in the male). The age of patients ranged between 27 and 86 years old, with an average age of 72 for women and 70 for men.

In detail, from the initial dataset of 251 patients who underwent adrenalectomy in our institution, we excluded from further analysis the following: 95 patients for missing histopathologic digital report; 85 patients due to missing NECT before surgery; 4 patients for cortical adrenal hyperplasia; 10 patients for infiltrating masses; 4 because of other benign conditions (i.e., hematomas, cysts); only 5 patients were ineligible because their NECT scans could reliably diagnose adenomas with a mean density lower than 10 HU.

The histopathological classification of the lesions was the following: 19 adenomas (38%), 9 pheochromocytomas (18%), 5 adrenal carcinomas (10%), 7 myelolipomas (14%), 8 metastases (16%), 1 mesothelioma, and 1 cavernous hemangioma (4%). Patients had an average lesion diameter of 5.5 cm with a minimum diameter of 1.5 cm and a maximum of 14.7. The characteristics of the patients and lesions included in the final analysis are summarized in Table 1.

**Table 1:** Characteristics of patients and lesions included in the final analysis

Patients (*n*)	48
Females; males (*n*)	22; 26
Age (average; range, years)	61; 27–86
Lesions (*n*)	50
Diameter (average; range, cm)	5.5; 1.5–14.7
Laterality	
Monolateral (*n*; %)	46; 92%
Bilateral (*n*; %)	4; 8%
Histology (*n*; %)	
Adenoma	19; 38%
Pheocromocytoma	9; 18%
Metastasis	8; 16%
Adrenal carcinomas	5; 10%
Myelolipoma	7; 14%
Other histology	2; 4%

Multivariable logistic regression on clinical, demographical, and radiological characteristics of the patients is shown in Table 2 demonstrating that none of these parameters are associated with the outcome.

**Table 2:** Multivariable logistic regression of clinical (age, sex) and standard radiological characteristics (maximum 3D diameter and mean HU). *HR* hazard ratio

	Multivariate logistic regression			
	*p* value	HR	95% C. I. of HR	
			Inf.	Sup.
Max3Ddiameter	0.189	1.230	0.903	1.673
MeanHU	0.900	1.002	0.976	1.028
Age	0.382	1.020	0.975	1.067
M/(F)	0.564	0.688	0.193	2.454

The 30 logistic regression models trained by LASSO resulted in an average AUC of 0.95 (0.81–1.00) (excluding repetitions when the algorithm did not reach convergence (5 times). On the test set, the models had an average AUC of 0.72 (0.48–1.00). The best-performing logistic regression model had an AUC of 0.99 in the training phase and 1.00 in the test phase and was composed of 13 features.

**Fig. 4:**
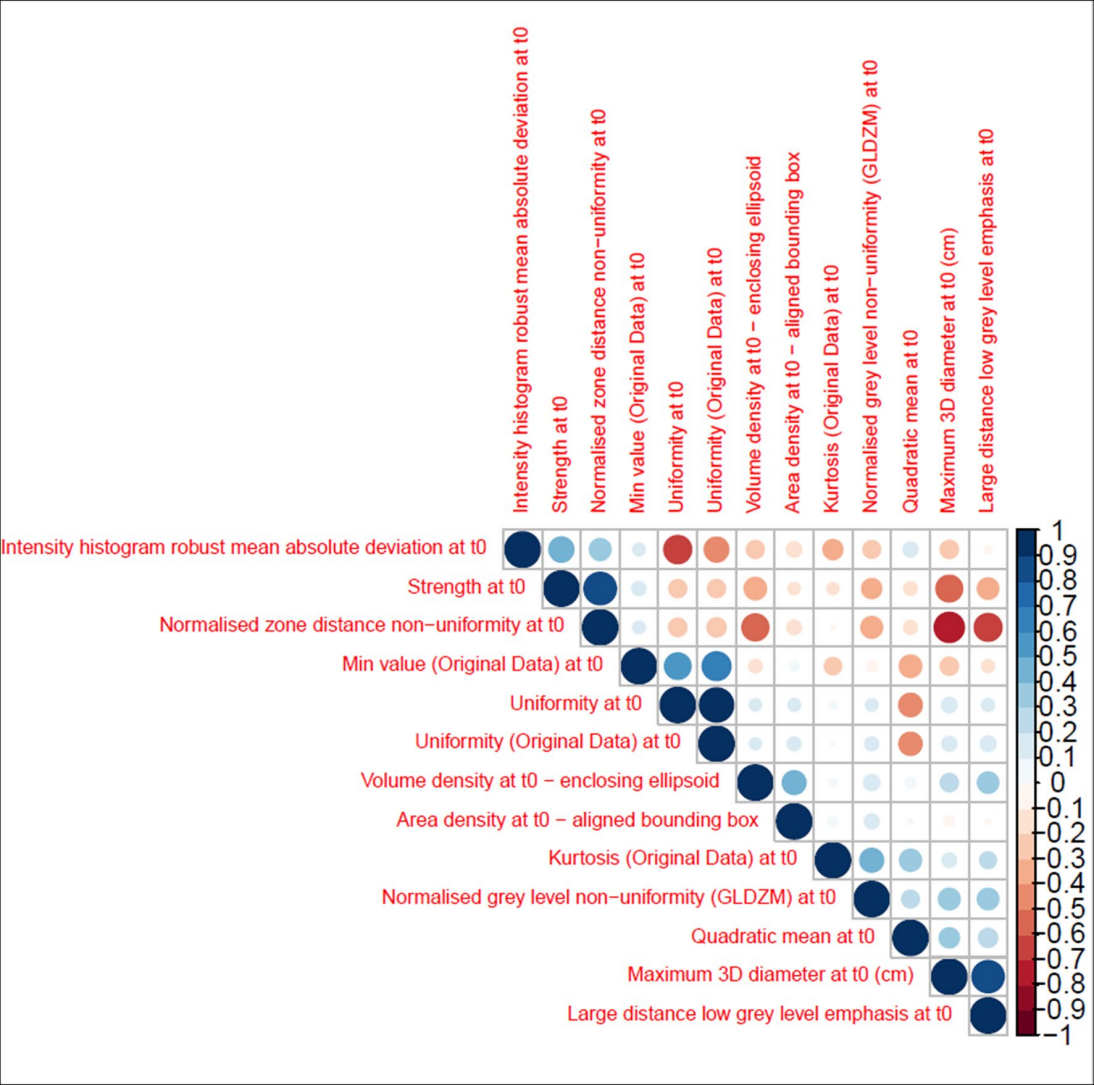
Pearson’s *ρ* correlation coefficient of the 13 features selected by LASSO in the best-performing model among the 30 iterations

In Fig. 4, we show Pearson’s correlation coefficient results and features with a Rho > 0.80 were eliminated. To prevent overfitting, only the top 5 informative features from the 30 LASSO iterations (quadratic mean, strength, maximum 3D diameter, volume density, and area density) were retained for further analysis.

**Fig. 5:**
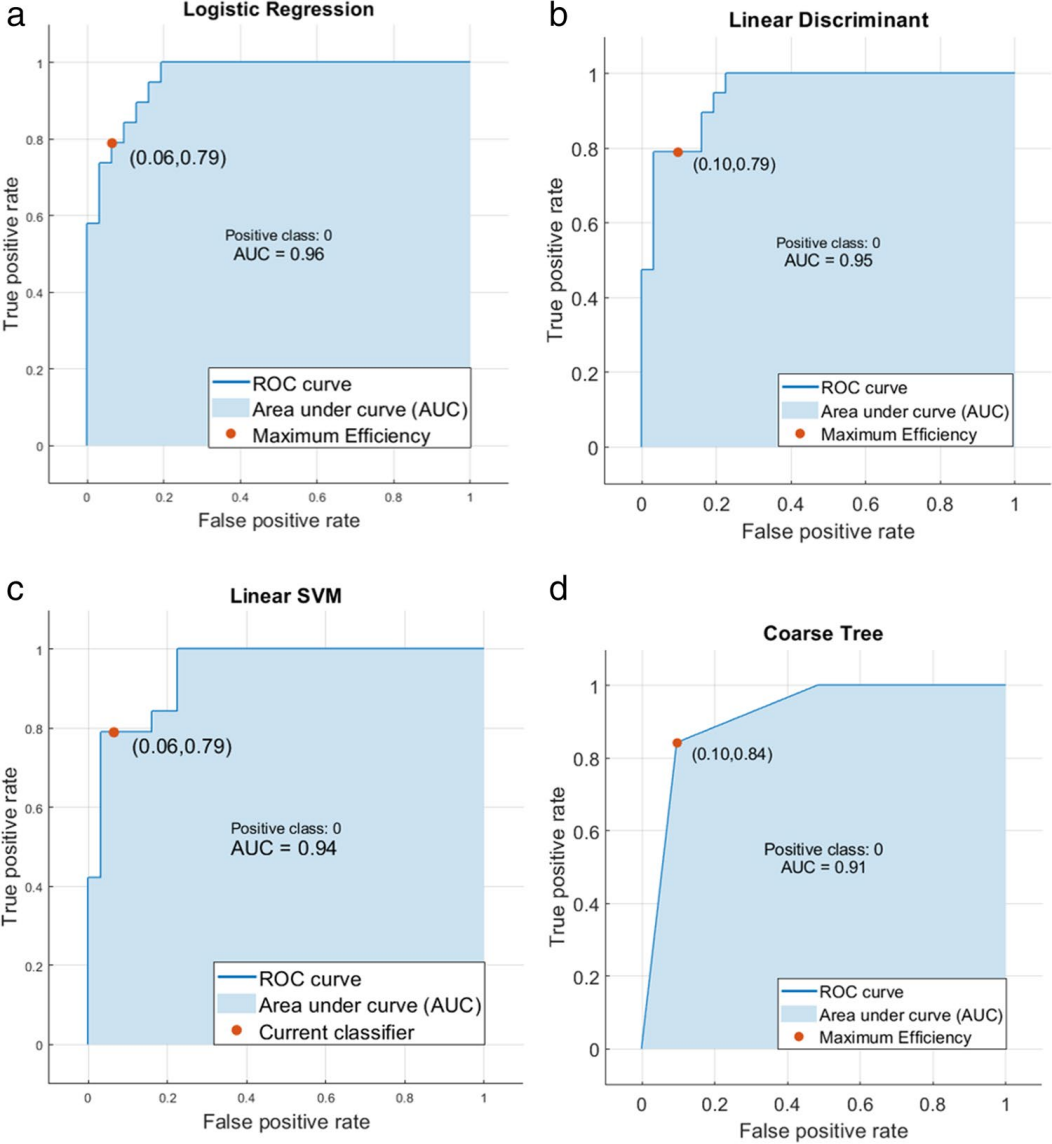
Model performances in terms of ROC curves and AUC for (**a**) logistic regression, (**b**) linear discriminant, (**c**) linear SVM, (**d**) coarse tree

**Fig. 6:**
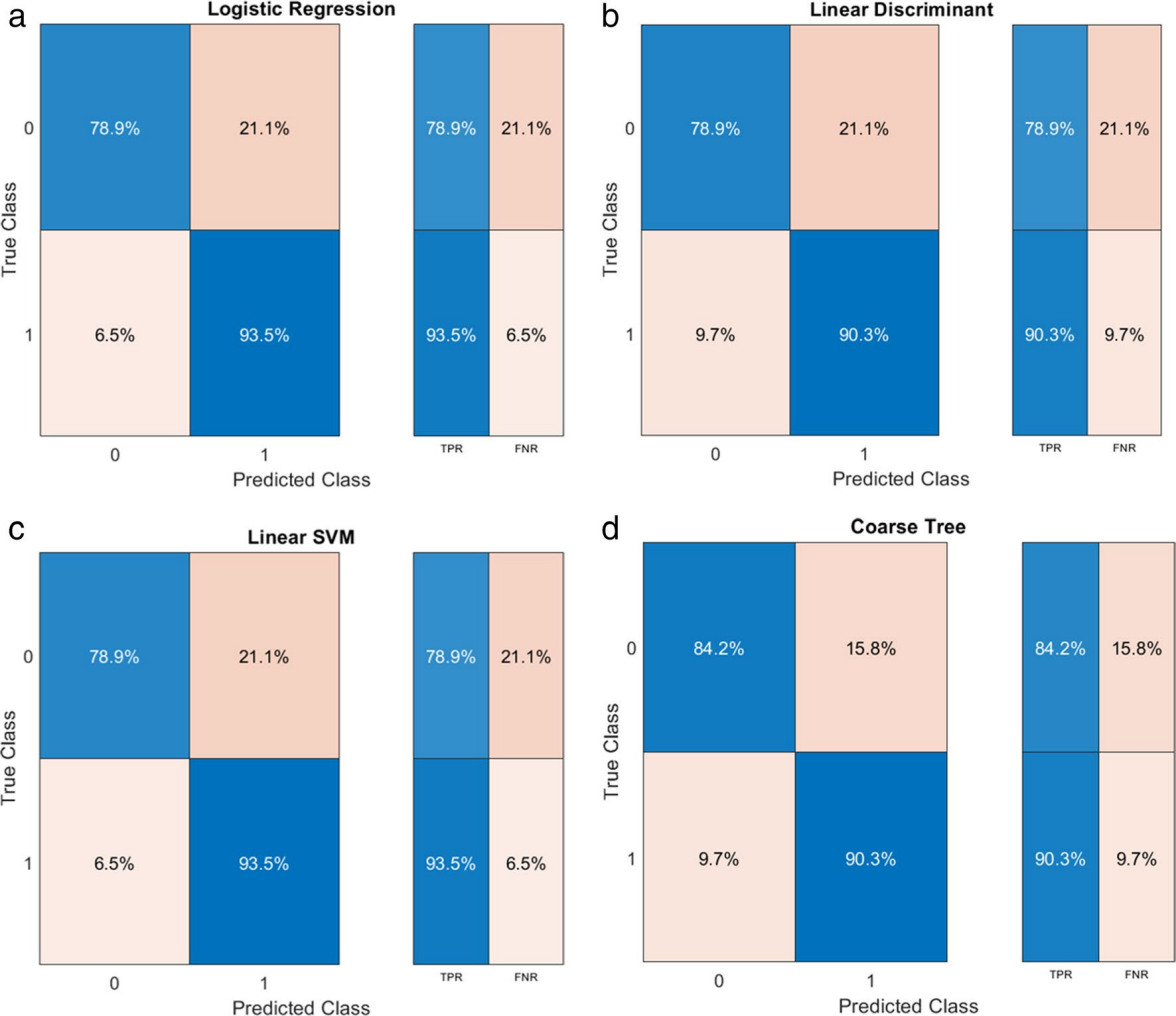
Model performances in terms of confusion matrices and TPR and FNR for (**a**) logistic regression, (**b**) linear discriminant, (**c**) linear SVM, (**d**) coarse tree

In the end, we trained the 4 final models on the entire lesion dataset employing logistic regression, linear discriminant, support vector machine, and decision tree as classifiers, obtaining an AUC of 0.95, 0.94, 0.91, and 0.96, as can be seen in Fig. 5. True positive rates and false negative rates and other classification performances can be appreciated in the confusion matrices of the models reported in Fig. 6. Calibration and decision curves of the logistic regression model with the comparison with standard clinical parameters are available as supplementary material 2.

## Discussion

Physicians’ desire for diagnostic certainty and, on the other hand, discomfort with diagnostic uncertainty when faced with an unexpected or unexplained imaging finding can lead to an increase in test ordering. As a result, further imaging and clinical evaluation are often performed when an adrenal incidentaloma is discovered and when imaging findings are equivocal or inconclusive [2, 21, 22].

In the present study, a radiomic signature composed of first- and higher-order features, namely quadratic mean, strength, maximum 3D diameter, volume density, and area density, showed a very good average performance with AUC = 0.94 (0.91–0.96) among the four final classifiers to discriminate adenomas from other adrenal lesions at NECT.

The performances of our machine learning models did not differ significantly, with logistic regression showing the best results with an AUC of 0.96 (Fig. 5a). The true positive rate for adenomas, according to the model, was 79%, whereas it was higher (93.5%) for non-adenomas, as shown in Fig. 6a. The decision tree performed better for both classes, with a true positive rate of 84.2% and 90.3% (Fig. 6d).

Our signature’s performance is comparable to that of the three models developed by Zhang et al [18] for differentiating lipid-poor adenomas using conventional, radiomic, and integrated conventional-radiomic CT features. These models had an AUC of 0.94, 0.93, and 0.96, respectively. However, in their cohort, conventional parameters such as gender, age, mean HU, and tumor diameter were strong predictors of the outcome at both univariable and multivariable logistic regression. As a result, their radiomic signature did not significantly improve the performance of the conventional model, i.e., employing standard radiological features and demographic data, thereby diminishing the scientific impact of their results.

Conversely, the net benefit of using our radiomic signature in comparison to standard parameters is clearly visible from the decision curves shown in supplementary figure 2. In our opinion, these differences are easily addressed by the different cohorts of patients used to train and test the models in the two studies, which may have led to some selection biases while considering broader inclusion criteria for eligible lesions in the aforementioned work. As shown in Table 2, indeed, no standard parameters in our cohort were statistically significant predictors of the outcome using multivariable regression.

The composition of the radiomic signature reveals additional distinctions. Indeed, our signature is composed of quadratic mean, strength, maximum 3D diameter, volume density, and area density. The quadratic mean is a first-order feature derived from a histogram that has a fair correlation to the HU median (or mean) value, which is found in the work of Zhang et al [18], Cao and Xu [15], and O’Shea et al [14] for the differentiation of lipid-poor adenomas from other histotypes.

Strength is a more complex second-order feature that is related to the texture of the image; in particular, it can be correlated to the concepts of coarseness, as specifically described by Amadasun and King [23]. In this context, a high strength means that the patterns that compose the texture of the tumor appear larger with broader areas of uniform pixel intensities whereas a low strength would correspond to a finer texture leading to higher variations in local pixel intensities. To our knowledge, this predictor has not been investigated in any other published study.

Maximum 3D diameter is a parameter already employed in clinical practice and reported in previous studies cited above. In the end, area and volume density are related to the shape and extent of the tumors and may provide additional information on their morphological appearance. In fact, adenomas present more frequently as well-demarcated round or oval lesions [9]. These two parameters likely reflect and quantify these visual characteristics of the tumor that were not quantified in previous studies or were only partially considered when the greatest or shortest diameters and tumor volume were used [15]. Our findings suggest that additional metrics, beyond the mere measurement of the mean density, should be considered for inclusion in routine radiological evaluation of adrenal lesions to reduce the number of incidentalomas regarded as indeterminate at NECT examination, thus avoiding unnecessary clinical workup and follow-up examinations.

At NECT, adenomas present more frequently with low attenuation (less than 10 HU) due to a microscopic fat component. This cut-off is highly specific (sensitivity 71%, specificity 98%) [4, 6], widely accepted in the scientific literature, and routinely employed in radiological practice [24, 25]. However, NECT alone is not always diagnostic, since 15–30% of adenomas are lipid-poor, namely containing insufficient intracytoplasmic lipid to conform to the non-contrast features previously described, thereby demonstrating higher attenuation values [26]. Previous works have shown that decreasing the HU threshold for the identification of adenomas could improve the specificity but reduce the sensitivity, whereas increasing such a threshold could result in improved sensitivity but reduced specificity [27, 28].

In a study by Yi and colleagues [17], aiming to differentiate histology-confirmed lipid-poor adenomas from pheochromocytomas, the authors built two radiomic nomograms using NECT and contrast-enhanced CT data, respectively, and concluded that the additional contrast-enhanced adrenal CT may not be necessary. Indeed, the drawbacks of a second scan can include additional costs, radiation risks, and potential harms associated with contrast media administration, including allergy and potential renal injury. A dedicated adrenal CT protocol including a 15-min delayed acquisition and considering a 60% threshold for contrast washout has been shown to properly classify 96% of adrenal masses, with 98% sensitivity and 92% specificity for discriminating adenomas from non-adenomas [29]. However, it should be noted that the additional role of dedicated CT protocols in characterizing incidental adrenal masses based on washout calculation has a low sensitivity and specificity in the literature, particularly in the case of suspected pheochromocytomas or metastases from hypervascular tumors which frequently demonstrate rapid contrast washout [22]. Hypervascular metastases from renal cell carcinoma and hepatocellular carcinoma are examples that may include intracellular lipids and have washout values similar to adenomas [30].

Furthermore, the patient is required to return for dedicated adrenal imaging if the initial NECT, in which the lesion had been incidentally detected, was inconclusive; this will obviously lengthen the diagnostic process and cause psychological distress to the patient.

There are several limitations to this study that should be considered. One major limitation is the small sample size of the final cohort. This was inevitable because our efforts to find patients who had adrenal nodules that were indeterminate at NECT, with the necessity of histological confirmation, resulted in a relatively small number of lesions meeting the inclusion criteria. Another limitation is the retrospective nature of the image data acquisition: in this observational study, the type of scanner used for each patient was not controlled. When considering the robustness of radiomic applications in the clinical setting, the potential impact of variation in CT data acquired from different scanners should not be understated. However, our radiomic signature is based on 1 histogram-based feature, 1 second-order feature, and 3 shape features that have been shown to be robust in previous radiomics studies [31, 32].

## Conclusions

Including additional imaging indicators for the identification of lipid-poor adenomas can increase the accuracy of NECT and reduce the need for additional imaging and clinical workup, according to this and other recent studies focusing on radiomics that have distinct points of contact with current clinical practice.

Our radiomic signature based on 1 histogram-based feature, 1 second-order feature, and 3 shape features could be considered for integration in routine radiological assessment of adrenal lesions, beyond the mere measurement of median density. This may serve as a method of enhancing the diagnostic power of NECT in order to substantially limit the number of adrenal incidentalomas initially regarded as indeterminate.

### Supplementary Information

Below is the link to the electronic supplementary material.

Supplementary file1 (ZIP 167 KB)

## Abbreviations

AUC: Area under the curve
IBSI: Image Biomarker Standardization Initiative
LASSO: Least absolute shrinkage and selection operator
NECT: Non-enhanced CT
ROC: Receiver operating curve
ROI: Region of interest
VOI: Volume of interest

## Declarations

### Guarantor

The scientific guarantor of this publication is Aldo Carnevale.

### Statistics and biometry

One of the authors has significant statistical expertise.

### Study subjects or cohorts overlap

No study subjects or cohorts overlap.

### Methodology

- retrospective
- observational
- performed at one institution
