# Supplementary material for: Radiomics in the characterization of lipid-poor adrenal adenomas at unenhanced CT: time to look beyond usual density metrics
Source: Eur Radiol. 2023 Aug 11;34(1):422–32. doi: 10.1007/s00330-023-10090-8 (PMC10791982; doi:10.1007/s00330-023-10090-8)
Supplement: Supplementary file 1 — Supplementary file1 (ZIP 167 KB) [file 330_2023_10090_MOESM1_ESM.zip › EURA-D-23-00828_ESM/EURA-D-23-00828_ESM.pdf]

Radiomics in the characterization of lipid-poor adrenal adenomas at unenhanced CT: time to look beyond usual density metrics

ELECTRONIC SUPPLEMENTARY MATERIAL

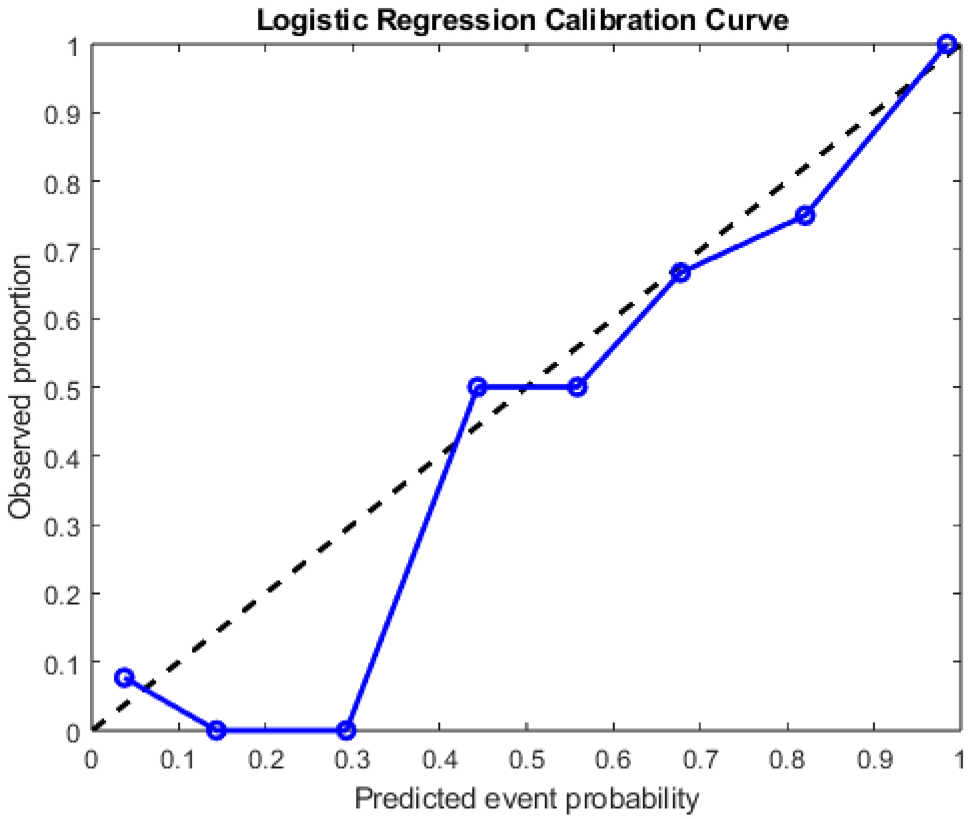

Supplementary Figure 1*: Calibration curve for the final Logistic regression model*

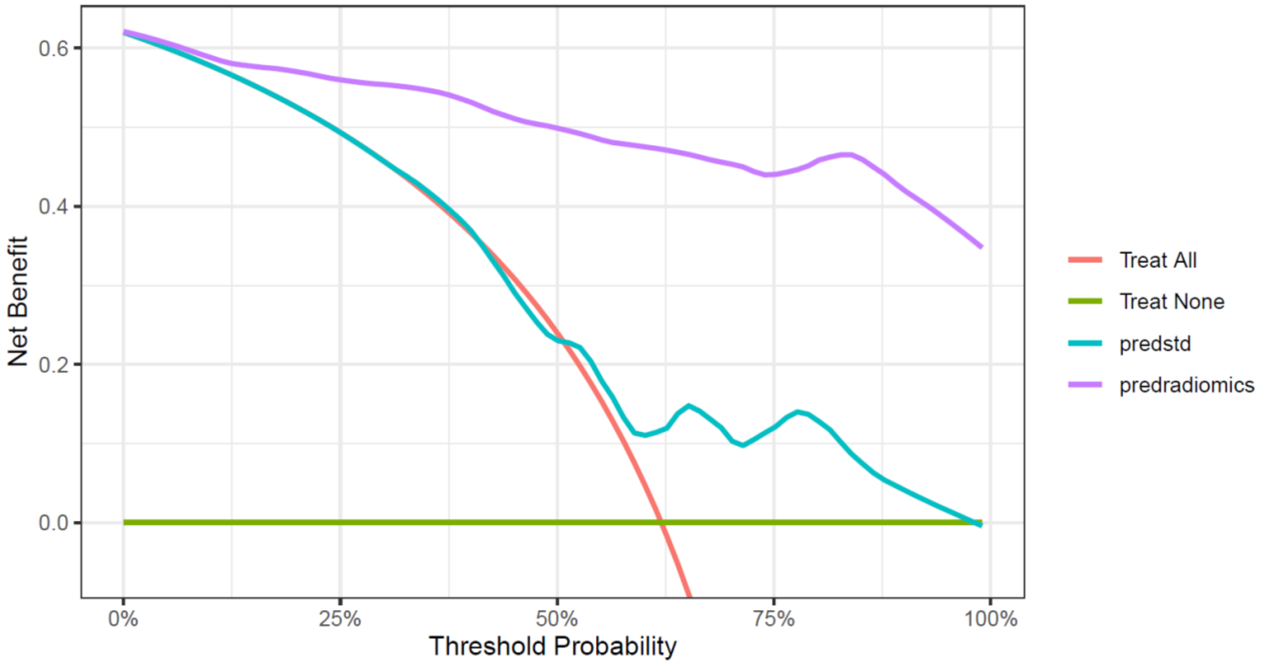

Supplementary Figure 2*: Decision Curve for the final radiomics based logistic regression model (predradiomics) and its comparison with prediction of standard clinical (Age, Sex)l and radiological (mean HU, Max diameter 3D) predictors (predstd)*
